# Structural delineation of stem-loop RNA binding by human TAF15 protein

**DOI:** 10.1038/srep17298

**Published:** 2015-11-27

**Authors:** Maruthi Kashyap, Akshay Kumar Ganguly, Neel Sarovar Bhavesh

**Affiliations:** 1 International Centre for Genetic Engineering and Biotechnology (ICGEB), Aruna Asaf Ali Marg, 110 067, New Delhi, India.

## Abstract

Human TATA binding protein associated factor 2 N (TAF15) and Fused in sarcoma (FUS) are nucleic acid binding proteins belonging to the conserved FET family of proteins. They are involved in diverse processes such as pre-mRNA splicing, mRNA transport, and DNA binding. The absence of information regarding the structural mechanism employed by the FET family in recognizing and discriminating their cognate and non-cognate RNA targets has hampered the attainment of consensus on modes of protein-RNA binding for this family. Our study provides a molecular basis of this RNA recognition using a combination of solution-state NMR spectroscopy, calorimetry, docking and molecular dynamics simulation. Analysis of TAF15-RRM solution structure and its binding with stem-loop RNA has yielded conclusive evidence of a non-canonical mode of RNA recognition. Rather than classical stacking interactions that occur across nitrogen bases and aromatic amino acids on ribonucleoprotein sites, moderate-affinity hydrogen bonding network between the nitrogen bases in the stem-loop RNA and a concave face on the RRM surface primarily mediate TAF15-RRM RNA interaction. We have compared the binding affinities across a set of single-stranded RNA oligonucleotides to conclusively establish that RNA binding is dependent upon structural elements in the RNA rather than sequence.

Human TATA binding protein associated factor 2N (TAF15/TAF2N), also known as RNA binding protein 56 (RBP56), belongs to a conserved FET family of proteins. Other members of this protein family include EWS (Ewing’s sarcoma) and FUS/TLS (fused in sarcoma/translocated in liposarcoma) proteins^1^. FET proteins are predominantly nuclear proteins^2^, but they have also been shown to shuttle between the nucleus and cytoplasm^3^,^4^,^5^ thus expanding their functional repertoire to DNA binding^6^, RNA processing events like pre-mRNA splicing and mRNA transport^7^, regulation^8^ and interaction with diverse number of proteins^9^.

TAF15 is normally found associated with RNA polymerase II in transcription pre-initiation complexes^1^ as well as with the U1 snRNP component of the spliceosome^10^, thus acting as a vital cog in the coupling of transcription and RNA processing. Mutations in the TAF15 gene have been implicated as potential cause of familial amyotrophic lateral sclerosis (FALS)^11^. Oncogenic alterations of TAF15 have been previously characterized in cases of acute leukemia, wherein gene translocations give rise to fusion protein of TAF15 N-terminal domain with other transcription factors such as CIZ/NMP4^12^. Similar fusion proteins have also been found for FUS and EWS in myxoid liposarcoma and neuroectodermal tumour cells, respectively^13^,^14^,^15^.

Architecturally, TAF15 is composed of an intrinsically unstructured N-terminal QGSY rich activation domain which is retained in the oncogenic fusions and possesses trans-activating properties^16^,^17^, followed by a central and the C-terminal region comprising the RNA recognition motif (RRM) and a (Cys)_4_ RanBP2 type Zinc finger (ZnF) with interspersed unstructured elements rich in arginine and glycine, known as RGG motifs. The RRM exhibits highest degree of sequence conservation among the members of the FET family.

FUS and TAF15 are functionally more related to each other than EWS^18^. The first report attributing an RNA binding function to FET protein family was identified in FUS, indicating it as a potential splicing regulator of E1A pre-mRNA and *β*-tropomyosin pre-mRNA with its C-terminal region having high affinities to GGUG sequence motifs^19^. Subsequent investigations indicated that the RRM plays no role in GGUG RNA recognition and the RanBP2 type zinc finger mediates the recognition^20^,^21^. Recent transcript analysis has provided contrasting insights into the importance of RNA secondary structural elements in recognition^22^,^23^,^24^. A consensus is yet to be reached and till date, despite these studies, the RNA binding preferences of this family still remains elusive. Considering that loss of RNA binding function by FUS and TAF15 is partly manifested as the neurodegenerative disorder ALS^11^,^25^, a comprehensive evaluation of the RNA targets is required.

TAF15, relative to its counterpart, is less characterized. TAF15 regulates gene expression of cell cycle regulatory genes through a pathway involving miRNAs^26^, is required for a critical alternative splicing event of the zeta-1 subunit of the glutamate N-methyl-D-aspartate receptor^27^, and associates with a part of human U1 small nuclear RNA^28^. PAR-CLIP analysis of all the human FET proteins, including TAF15, indicated that all the FET proteins bind to a *SON* (stem in natural left right configuration) stem-loop cluster with higher affinities than that for RNA with GGU repeat^29^.

Though there is plethora of literature pertaining to the functional roles of FET protein family and their implications in various disease conditions, a thorough biophysical or structural characterization is still lacking. This can be possibly attributed to its high intrinsic disorder making it not easily amenable for such studies. So far, only the RRM domain of FUS has been structurally characterized^30^. While the role of N-terminal region has been explained^31^ the functional role of C-terminal region is still debatable. This study provides a comprehensive biophysical and structural analysis centered on the RRM, RGG motif and the RanBP2 type zinc finger of TAF15, which forms a part of its C-terminus, and provides molecular glimpses into GGUG, stem-loop and CUG containing RNA binding events in solution.

## Results and Discussion

### RRM and the RanBP2 type ZnF domains of TAF15 do not interact with each other

We previously reported the sequence specific resonance assignments of TAF15-RRM and TAF15-RRM-RanBP2^32^. Detailed analysis of the dynamics of entire polypeptide chain of TAF15-RRM and TAF15-RRM-RanBP2 was investigated by ^15^N-{^1^H} Het-NOE measurements. ^15^N-{^1^H} Het-NOE values, corresponding to an average of ~0.75 in the RRM and ZnF domains, and ~0.38 in the RGG linker, indicated the presence of highly dynamic linker between the structured RRM and the RanBP2 type ZnF domain in the multi-domain protein (Supplementary Fig. S1). Moreover, random coil index analysis (RCI) of the backbone^32^ revealed the absence of any secondary structural elements between E323-K354, which constitute the RGG motif. All these observations along with sharp spectral line widths in 2D [^15^N,^1^H] HSQC spectra^32^ conclude that the RRM and the RanBP2-type ZnF domains do not interact with each other.

### TAF15-RRM adopts a canonical RRM fold and has an extended α_1_-β_2_ loop

**Figure 1:**
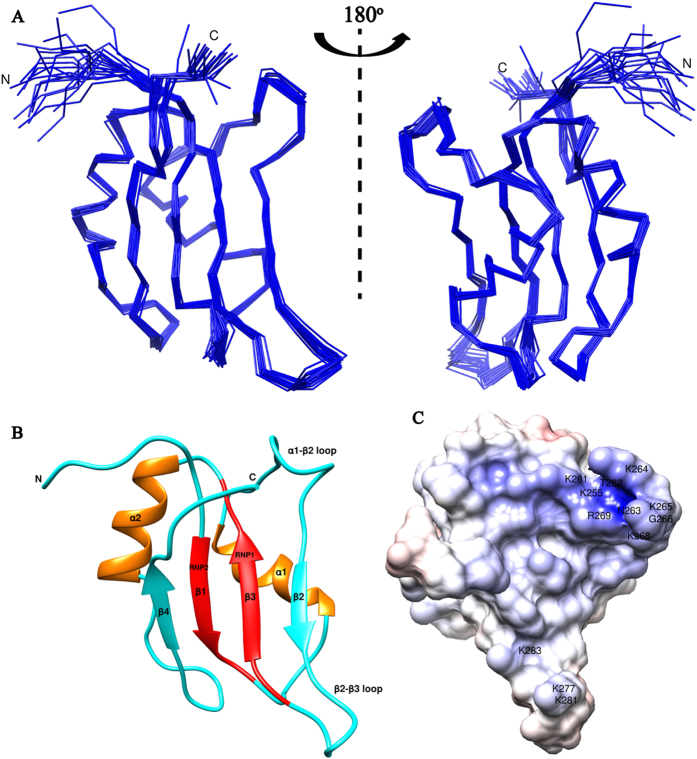
(**A**) C^α^ trace of 20 lowest energy conformers of TAF15-RRM (**B**) Cartoon representation of TAF15-RRM. N and C represent the termini. Highly conserved RNP2 and RNP1 sites are colored red. Prominent loops are indicated. **(C)** Electrostatic potential surface of TAF15-RRM highlighting the positively charged cluster (labeled). The surface is colored blue for potentials >10 kT/e and red for potentials <−10 kT/e.

An ensemble of 20 lowest energy minimized structures had a global backbone RMSD of 0.8 Å and an all atom RMSD of 1.2 Å (Fig. 1A). Further, structured regions had a backbone RMSD of 0.3 Å and an all atom RMSD of 0.7 Å. These results indicate a highly converged structure ensemble with minimal global conformational heterogeneity. A full summary of structural statistics is tabulated in Table 1. In agreement with RCI values^32^, solution structure revealed that TAF15-RRM has a canonical RRM fold (*β*_1_*α*_1_*β*_2_*β*_3_*α*_2_*β*_4_) consisting of four anti-parallel *β*-strands and two *α*-helices arranged as an *α*/*β* sandwich. T235-Q239 forms the *β*_1_, I271-T275 forms the *β*_2_, P282-S289 forms the *β*_3_ and K314-F317 forms the *β*_4_ strands. *β*_1_ and *β*_2_ strands are connected by *α*_1_-helix (T247-Q256) while *β*_3_ and *β*_4_ strands are connected by *α*_2_-helix (P293-F303). The structure contains several loops; loop L1 (G240-S246) between *β*_1_ and *α*_1_, loop L2 (I257-I271) between *α*_1_ and *β*_2_, loop L3 (D276-K281) between *β*_2_ and *β*_3_, loop L4 (F290-P293) between *β*_3_ and *α*_2_ and loop L5 (D304-I313) between *α*_2_ and *β*_4_. The two highly conserved ribonucleoprotein (RNP) sequence motifs, namely RNP2 (I236-L241) and RNP1 (K283-F290); are located on *β*_1_ strand and proximal part of *β*_1_-*α*_1_ loop, and on *β*_3_ strand and distal part of *β*_2_-*β*_3_ loop, respectively.

Interestingly, only two aromatic residues, F237 and F290, are present in the RNP2 and RNP1 sites of TAF15-RRM (Supplementary Fig. S2A), which are otherwise anywhere between 3–5 in case of most other canonical RRMs. Among these two aromatic residues, the aromatic ring of F290 on the RNP1 motif is oriented towards the structural core, indicating a much larger role played by it in providing structural strength rather than engaging in RNA recognition. The only stacking interaction arising from the RNP sites maybe provided by the surface exposed aromatic side chain of F237 on the RNP2 motif. Therefore, it is likely that these two sequence motifs might not play a much prominent role in RNA recognition. One unique structural feature of TAF15-RRM is its extended *α*_1_–*β*_2_ loop, which is much less predominant in case of other RRMs (Fig. 1B). RRMs have been shown to generate extreme functional diversity through their variable loops^33^, hence it is likely that the extended *α*_1_-*β*_2_ loop of TAF15-RRM may play a much larger role in RNA recognition.

Electrostatic surface analysis of TAF15-RRM reveals a dense positively charged cleft formed by the *α*_1_-*β*_2_ loop (Fig. 1C). Structural alignment of TAF15-RRM with other members of the FET protein family revealed that the extended *α*_1_-*β*_2_ loop is conserved in all the members of FET protein family, which is otherwise truncated in many other RRMs (to name a few, RRMs of ETR3 [4LJM], CstF [1P1T] and U2AF65 [2YH1]). The absence of significant number of surface exposed aromatic side chains in the *β*_1_ and *β*_3_ strands and the presence of an extended *α*_1_-*β*_2_ loop in all the members of FET protein family (Supplementary Fig. S2B) possibly indicate a unique mechanism adopted by this protein family in recognizing their cognate RNAs. Also, this high structural convergence indicates that the RRM domain of FET protein family may exhibit similar functional preferences.

### RRM predominantly mediates binding of TAF15 and FUS to *SON* stem-loop RNA

RNA targets for the FET family of proteins have been identified recently, which highlight the importance of RNA secondary structure in recognition, specifically the AU-rich stem loops and the *SON* (stem in natural left-right configuration) cluster^24^,^29^. However, the RNA binding regions in either of the members of the FET protein family involved in *SON* stem-loop RNA recognition is still unknown. To determine the RNA binding regions, different constructs of TAF15 and FUS expressing the RRM alone (TAF15-RRM, FUS-RRM) and RRM along with the disordered RGG motif and RanBP2 type zinc finger (TAF15-RRM-RanBP2, FUS-RRM-RanBP2) were analyzed for binding using ITC and solution-state NMR spectroscopy.

Structural integrity of the *SON* stem-loop RNA was confirmed by measuring the 1D ^1^H spectrum in water, which showed 8 imino proton resonances between 10.9 and 14 ppm indicating eight hydrogen bonded nucleotide pairs, corresponding to the stem region.

To gain insight into the Protein-RNA interaction isothermal titration calorimetry was performed. The equilibrium binding measurements revealed similar binding affinities of TAF15-RRM (*K*_*d*_ = 10 μM), TAF15-RRM-RanBP2 (*K*_*d*_ = 6 μM), FUS-RRM (*K*_*d (avg)*_ = 11 μM) and FUS-RRM-RanBP2 (*K*_*d*_ = 8 μM) with the *SON* stem-loop RNA (Supplementary Fig. S3). Thermodynamic parameters obtained from the fitted curves are indicated in Table 2.

**Figure 2:**
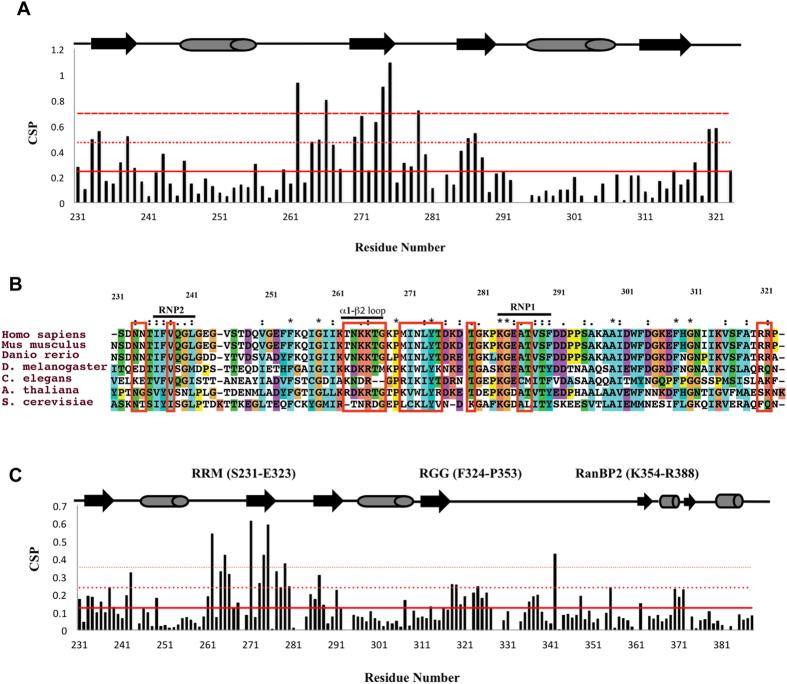
Chemical shift perturbation profile (CSP) of (**A**) TAF15-RRM and (**C**) TAF15-RRM-RanBP2 upon binding to 1.2 molar equivalents of *SON* stem-loop RNA. The solid, dotted and dashed horizontal red lines indicate averaged CSP, one and two standard deviations (σ) from the averaged CSP respectively. The secondary structural elements are shown on the top (thick arrows indicate *β*-strands, cylinders indicate helices and thick line segments indicate loops and the disordered regions). **(B)** Sequence alignment of TAF15-RRM with homologs from different distantly related species. Regions enclosed in red boxes correspond to the residues of TAF15-RRM undergoing significant CSPs upon binding with *SON* stem-loop RNA.

The residues possibly involved in interaction with RNA were identified using backbone amide chemical shift perturbations (CSP). Upon titrating TAF15-RRM against *SON* stem-loop RNA, several residues showed significantly higher CSPs compared to the rest (Supplementary Fig. S4). Significant CSPs (residues exhibiting CSPs above the averaged CSP plus one standard deviation) were observed for the residues; N233-N234, V238, T262-G267, M270-T275, T279, A286-T287 and R320-R321. Highest CSP was observed for T275 and two major clusters on the RRM (T262-G267 and M270-T275) (Fig. 2A). The residues T262, T266, Y274, T275 and T279 exhibited CSPs more than two standard deviations above the mean. Sequence alignment of TAF15-RRM with different distantly related species revealed that the residues showing higher CSPs, especially the threonines and tyrosine, among others, which exhibit highest CSPs, are highly conserved indicating their possible conserved role in stem-loop RNA recognition (Fig. 2B).

To conclusively establish the predominant role played by RRM in mediating RNA binding and negate any significant contributions toward RNA recognition by the RGG motifs and the RanBP2 type zinc finger, TAF15-RRM-RanBP2 was titrated against increasing molar ratios of *SON* stem-loop RNA (Supplementary Fig. S4B). No further chemical shift perturbation for the backbone amide resonance of the protein was observed beyond a molar stoichiometric ratio of 1:1 of TAF15-RRM-RanBP2 and the RNA, indicating a single RNA binding site in the protein, in agreement with the ITC analysis. Maximum chemical shift perturbations were seen in the region corresponding to RRM (in agreement with the CSPs exhibited by RRM construct alone). Very few residues in the RGG motif (E323-R326 and G342), and the RanBP2 type zinc finger domain (S355, F370-R372) exhibited CSPs above the averaged CSP + 1σ (Fig. 2C). The CSPs in E323-R326 region can be explained from its existence as an extension of the C-terminal region of the RRM, which may provide additional contributions in stabilizing the complex. Despite these additional contributions, N- or C-terminal extensions in an RRM have been rarely shown to provide specificity in recognition.

### *SON* RNA interacts with TAF15-RRM *via* its loop

Partial sequence-specific resonance assignments of *SON* stem-loop RNA were obtained using a combination of 2D [^1^H,^1^H] NOESY and 2D [^1^H,^1^H] TOCSY as well as 2D [^13^C,^1^H] HSQC and 2D [^15^N,^1^H] HSQC spectra at natural abundance. Unambiguous resonance assignments of several resonances of paired nitrogen bases of the stem were obtained using a sequential ‘walk’ between adjacent uracil H3 and guanine H1 resonances (Supplementary figure S5A). Subsequently, cross-peaks between the imino region and base H^Aro^ protons were used to unambiguously identify purine H8, pyrimidine H6 and H5 and adenine H2 resonances in the stem. In addition, amino protons (-NH_2_) could be identified for several hydrogen-bonded bases. Assignments were confirmed using the increased resolution offered by 2D [^13^C,^1^H] HSQC and 2D [^15^N,^1^H] HSQC spectra. Resonances arising from loop protons were highly overlapped and exhibited broad resonance lines in both free and bond state. These resonances were left unassigned after assignment of H6, H8, H2, H5 and H1′ resonances of bases in the stem belongs to loop region.

**Figure 3:**
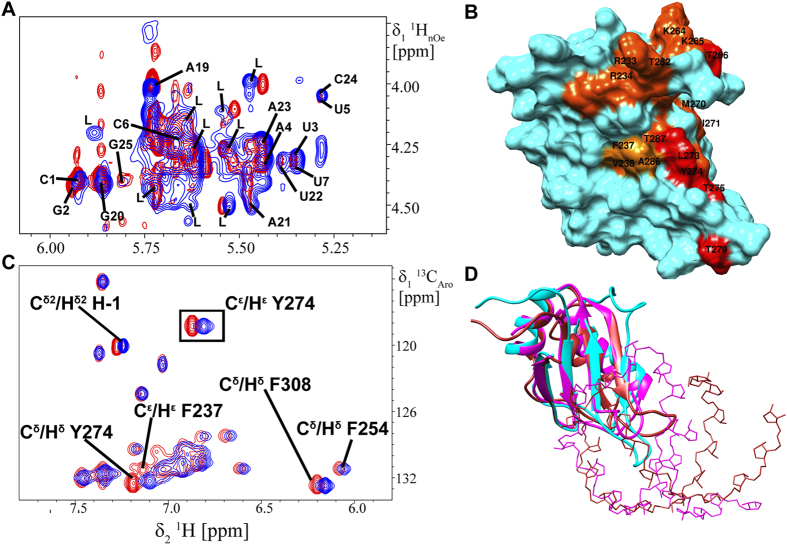
(**A**) Overlay of 2D [^1^H,^1^H] NOESY spectrum of free (red) *SON* RNA and 2D ^13^C-[ω_1_,ω_2_]-filtered [^1^H,^1^H] NOESY (*t*_mix_ = 350 ms) in D_2_O at 298 K of *U*-^13^C,^15^N TAF15-RRM with unlabeled RNA (blue) showing changes in intra-ribose H1′-H2′ cross-peaks at 298 K. Peaks arising from the RNA loop are labeled ‘L’. **(B)** Mapping of CSPs induced by *SON* stem-loop RNA binding to TAF15-RRM. The color scheme is; orange-red and red for one and two standard deviations from the averaged CSP respectively. **(C)** Chemical shift changes of H^δ^/C^δ^ and H^ε^/C^ε^ resonances of Y274, H^ε^/C^ε^ of F237 and other aromatic residues perturbed to a lesser extent upon RNA binding, shown in an overlay of 2D [^13^C_Aro_,^1^H_Aro_] HSQC spectrum of unbound (red) and 1:1.2 bound TAF15-RRM (blue) with *SON* stem-loop RNA at 298K. **(D)** Structural alignment of TAF15-RRM (PDB ID 2MMY; cyan) with RBMY (PDB ID: 2FY1; brown) and U1A (PDB ID: 1URN; magenta) stem-loop RNA complexes.

In order to identify resonances involved in the interaction with TAF15-RRM comparisons were drawn between *SON* resonances in bound and unbound states. Highest perturbations were observed for H1′-H2′ cross-peaks of loop resonances (Fig. 3A), indicating that the RNA backbone of the loop region was highly perturbed upon complex formation. Conversely, all imino proton resonances of the stem were found to remain unperturbed, barring changes in peak intensity, possibly arising due to fluctuations in chemical exchange/solvent accessibility (Supplementary figure S5B). This was further reflected in the absence of chemical shift perturbations for cross-peaks between imino protons and H6, H5, H8 and H2 protons of nitrogen bases (Supplementary figure S5C), conclusively eliminating the role of the stem in the interaction with TAF15-RRM. Additional perturbations were observed in a few intra-base and base-sugar cross peaks (pyrimidine H6-H5 and H6-H1′ and purine H8-H1′) belonging to the loop (Supplementary figure S5D).

### The concave face of TAF15-RRM forms the primary RNA binding interface

Residues of TAF15-RRM showing significant CSPs upon interacting with the *SON* stem-loop RNA were mapped on to the solution structure to define the possible binding interface (Fig. 3B). Interestingly, residues showing pronounced CSPs were localized to the concave face of the RRM, which is, distal end of *α*_*1*_*-β*_*2*_ loop (loop L2), *β*_*2*_ strand and the proximal part of *β*_*2*_*-β*_*3*_ loop (loop L3). This region appears to forms the primary binding interface, mainly because, all the residues that exhibit very strong CSPs (averaged CSP + 2σ) are confined to this region indicating a much larger role played by these loops and *β*_*2*_ strand in recognizing the RNA. Interestingly, all the threonines that showed maximum CSPs are part of this region, indicating that they play a crucial role in recognition through possible extensive H-bonding with the *SON* stem-loop RNA.

The general mode of RNA recognition by an RRM is through extensive base stacking interactions with the aromatics at highly conserved RNP2 and RNP1 sites, located on the *β*_*1*_ and *β*_*3*_ strands respectively (PDB IDs: 1UP1, 1CVJ, 1FXL)^34^,^35^,^36^. Surprisingly, as indicated by the lack of significant CSPs barring the exception of V238 (on the *β*_*1*_ strand, RNP2) and A286-T287 (on *β*_*3*_ strand, RNP1), it looks like the two RNP sites in TAF15-RRM play a less prominent or no role in *SON* stem-loop RNA recognition possibly due to the absence of critical aromatic residues. The N- and C-termini comprising the residues N233-N234 and R320-R321 showing significant CSPs also appear to contribute to binding. The significant CSP of C^δ^/H^δ^ and C^ε^/H^ε^ resonances of surface exposed aromatic side-chains of Y274 on *β*_*2*_ strand and F237 on *β*_*1*_strand (Fig. 3C) indicate the likelihood of their involvement in stacking interactions with RNA bases. Other aromatic residues perturbed to a lesser extent such as F308 on the *α*_*2*_*-β*_*4*_ loop and F254 on *α*_*1*_ helix were situated away from the binding interface and showed backbone amide CSPs lower than the mean (Fig. 2A). Their peak shifts were a probable result of minor rearrangements of the structured core upon RNA binding. Electrostatic surface analysis identified largely positively charged and few hydrophobic clusters wherein the aforementioned residues undergoing significant CSPs are confined to, providing an expanded positively charged surface for RNA binding. These emphasize the larger contribution of electrostatic and H-bonding interactions in mediating the *SON* stem-loop RNA binding to the RRM.

Though RRMs are predominantly *ss*-DNA/RNA binding proteins, there are structural reports on RRM-stem-loop complexes, with only four structures determined till date^34^,^37^,^38^,^39^. Structural analysis of three of these complexes (U1A, U2B” and RBMY) throws light on the significance of *β*_*2*_*-β*_*3*_ loop in mediating stem-loop RNA recognition (Fig. 3D). This loop, as well in TAF15-RRM, shows marked CSPs suggesting its role in binding. However, the unique feature of TAF15-RRM is its expanded *α*_*1*_*-β*_*2*_ loop, which is diminished or absent in either of the reported complexes. This unique expanded loop forming a dense positively charged cluster plays an important role in recognizing the stem-loop RNA, as evidenced by strong CSPs, suggesting a unique mechanism might be adopted by the TAF15-RRM in stem-loop RNA recognition.

### Backbone dynamics of TAF15-*SON* stem-loop RNA complex

The binding of *SON* stem-loop RNA to TAF15-RRM had no significant changes in the overall dynamics of the complex with respect to free RRM on the nanosecond-picosecond time scale (Supplementary Fig. S6A). Further, analysis of the regions exhibiting CSPs showed no significant changes with respect to dynamics suggesting that no significant structural ordering takes place either in the overall RRM fold or the loops upon complex formation with the RNA. To investigate the effect of complex formation on the distal RGG linker and RanBP2 type zinc finger domain, the backbone dynamics of TAF15-RRM-RanBP2-*SON* stem-loop RNA complex was analyzed (Supplementary Fig. S6B). There were again no significant changes in ^15^N-{^1^H} Het-NOE values pertaining to RGG linker or the RanBP2 type ZnF domain upon complex formation. Together with the CSPs and sharp line-widths observed in 2D [^15^N,^1^H] TROSY spectrum of TAF15-RRM-RanBP2 complex^32^, these results demonstrate that RRM and the RanBP2 type ZnF domains tumble independently in solution without any domain interaction, even after formation of the complex. These observations from the different experimental analyses suggest the absence of any domain-domain interactions in mediating RNA binding and substantiates that RRM alone is sufficient and plays a critical role in *SON* stem-loop RNA recognition.

### Unique recognition mechanism of *SON* RNA binding to TAF15-RRM

Given the extensive line-broadening and spectral overlap of resonances arising from the loop of *SON* RNA, unambiguous resonance assignment was not possible for this region despite our best efforts to optimize spectral parameters, temperature and buffer conditions. Moreover, inter-molecular NOEs were highly broadened to noise levels and could not be relied upon to derive distance restraints. As a result, the protein-RNA complex proved not amenable to conventional solution structure calculations. However, the chemical shift perturbations of non-stem resonances (Fig. 3A, supplementary figure S5D) provided sufficient evidence to suggest that the loop is directly involved in binding to TAF15-RRM. Additionally, our analysis of existing RRM-stem-loop RNA structures suggested that the RNA interacts with its cognate protein interface through bases on its loop region, which possess a higher degree of structural flexibility required for bases to adopt binding-friendly conformations. Given that the interaction was evident from isothermal titration calorimetry as well as perturbation mapping of TAF15-RRM residues, a soft-docking of *SON* RNA on to the RRM was performed using the CSP restraints and the resultant structure further improved using explicit solvent energy minimization, molecular dynamics and cluster analyses of trajectories.

**Figure 4:**
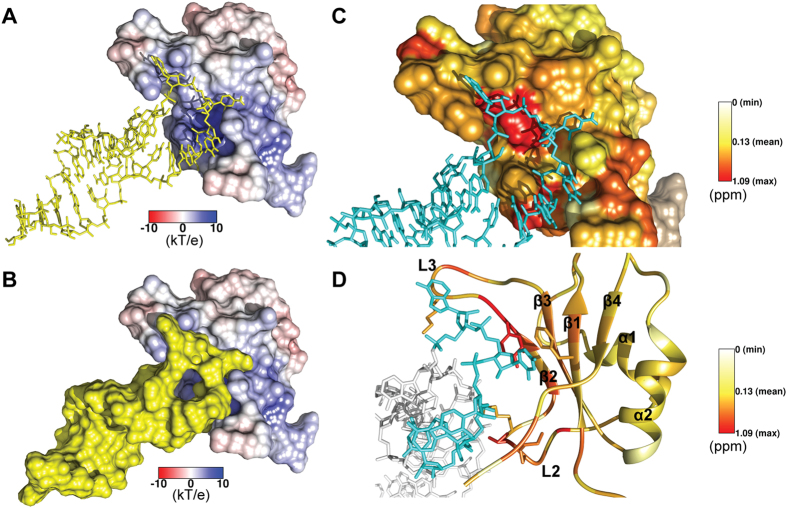
Structural model of TAF15-RRM-*SON* stem-loop RNA complex. The High Ambiguity Driven Protein-Protein Docking (HADDOCK) generated model using NMR structure of TAF15RRM (PDB 2MMY) was refined with molecular dynamics simulation (MD) in GROMACS with AMBER99SB-ILDN force field **(A)** Stick and **(B)** surface representations of RNA (yellow) on electrostatic surface of TAF15-RRM showing a positively charged (blue) cavity. **(C)** Surface and **(D)** ribbon representations of TAF15-RRM with CSP values mapped on residues, in complex with RNA (cyan). RNA binding is predominantly across the L2-*β*_2_-L3 interface as opposed to the entire *β*-sheet.

The docking of TAF15-RRM and *SON* stem-loop RNA was performed with High Ambiguity Driven Protein-Protein Docking (HADDOCK) using the experimentally derived CSP information that provided initial clues as to the role of loops L2 and L3 as well as a portion of the *β*_*2*_ strand in RNA recognition. The lowest energy docked structure after a 10 ns molecular dynamics simulation showed a 591 Å^2^ net surface area of the RNA loop (A11-A14) stably docked into a 564 Å^2^ positively charged cavity formed by K264, K265 and K268 on loop L2, K277, K281, K283 on loop L3, *β*_*2*_ and *β*_*3*_ strands as well as R320 and R321 on the carboxy terminus (Fig. 4A,B). As given in docking restraints, the majority of interfacial residues correspond to those with CSP greater than one standard deviation from the mean (Fig. 4C,D). Gross structural changes occurring on the TAF15-RRM and on the *SON* stem-loop RNA upon binding revealed a small inward folding (3.5 Å) of the L2 loop and a larger, outward movement of L3 (6.0 Å), along with minor rearrangements of F237 and Y274 aromatic rings and the *α*_*2*_ helix (Supplementary Fig. S7A). The bases in the loop region of *SON* stem-loop RNA underwent substantial rearrangement with A11 and C12 flipping outwards and the backbone in general adopted a more extended conformation (Supplementary Fig. S7B).

**Figure 5:**
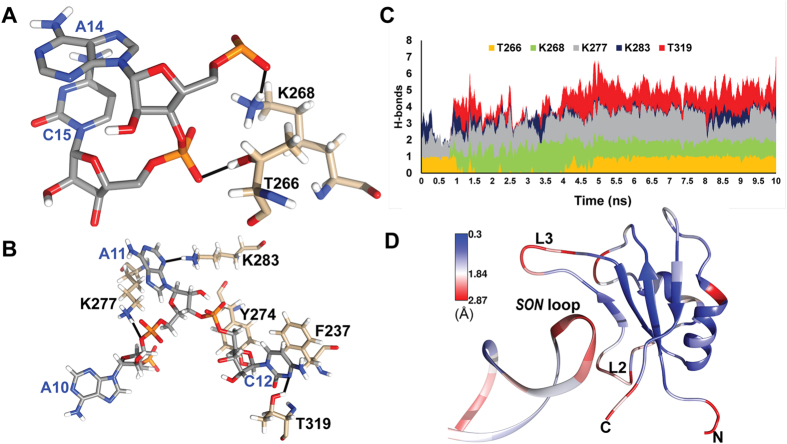
Interactions on RRM-RNA interface mainly mediated by hydrogen-bonding (shown as solid black lines) with the extended loop L2 (**A**), loop L3 and T319 on the carboxy terminus tail (**B**). Panel B also shows F237 and Y274 as the only two stacking amino acids. **(C)** Stacked bar graph showing hydrogen bonds between select interfacial amino acids and the *SON* loop through 10 ns of simulation time. **(D)** Ribbon representation of the complex colored by root mean squared fluctuation across the simulation, showing a high RMSF (Å) for A10-C15 on the *SON* loop and loops L2 and L3 on TAF15-RRM, despite a robust H-bonding network.

The complex is held in place *via* hydrogen bonds between N^ζ^/H^ζ^ of K277 and O3′ of A10, N/H of K277 and N3 of A11, N^ζ^/H^ζ^ of K283 and N3 of A11, O^γ^/H^γ^ of T319 and O2 of C12, N/H of T319 and O2 of C12, N^ζ^/H^ζ^ of K268 and O1P of A14 and O^γ^/H^γ^ of T266 with O1P of C15 (Fig. 5A,B). This hydrogen-bonding network was found to be stable after 5 ns of simulation (Fig. 5C). The hydrogen bond between K283 and A11 was largely transient, existing in sporadic 1 ns intervals. In cases where this bond was absent, the backbone amide proton of K277 adopted its role, ensuring that a net total of 5–7 redundant interfacial hydrogen bonds were always maintained. The H-bonding with the L2 loop interface was relatively static after 5 ns of the simulation, suggesting a crucial role-played by T266 and K268 in tethering the RNA loop. Surprisingly, barring T266 and Y274, the residues involved in H-bonding showed CSP values below mean + 2σ, signifying that their backbone amides remain largely unperturbed during complex formation.

Further anchorage was provided to the *SON* loop by an anion-π and CH-π stack between phosphate and ribose of C12 respectively, with the aromatic ring of Y274, as well as a displaced parallel π-π stack between C12 and F237 (Fig. 5B). The distances between centers of geometry for C12-PO_4_-Y274 and C12-ribose-Y274 were 4.1 and 3.9 Å, respectively. The π-π stack had an inter-planar distance of 3.3 Å and an angle of 5.3°. The multiple modes of binding to C12 led to a large buried surface area of 192 Å^2^ for C12 in the complex. Similarly, A11 (152 Å^2^), U13 (76 Å^2^) and A14 (104 Å^2^) exhibited high solvent inaccessibility. Protein residues that underwent major dehydration were F237 (46 Å^2^), T266 (36 Å^2^), K268 (48 Å^2^), Y274 (57 Å^2^), K277 (89 Å^2^), K283 (40 Å^2^) and T319 (82 Å^2^). Despite the robust nature of this interface, loops L2 and L3, along with A11-C15 of the RNA showed relatively high root mean squared fluctuations (RMSF) in the simulation (Fig. 5D), which is in agreement with ^15^N-{^1^H} Het-NOE data of RNA bound TAF15-RRM. The high redundancy of the hydrogen-bonding network allowed this dynamism on the nanosecond time scale by permitting repeated make and break events to occur between bond donors and acceptors.

### Structural elements in RNA are instrumental for recognition

Widespread studies on the FET protein family have revealed a number of RNA targets. The importance of RNA secondary structural elements in mediating recognition has been debatable till date, and a consensus is yet to be reached. In an attempt to highlight the importance of RNA secondary structural elements in mediating binding to TAF15-RRM, varying lengths of GGUG and CUG containing ssRNAs were extensively analyzed for binding using solution-state NMR spectroscopy.

A 6-mer RNA containing the GGUG motif (5′ GGUGUG 3′) was analyzed for binding to TAF15-RRM using CSPs from solution-state NMR spectroscopy (Supplementary Fig. S8A). Only few residues in the RRM showed significant CSPs (CSP + 1σ) upon binding to the RNA manifesting as part of N- and the C- termini along with the *β*_*2*_*-β*_*3*_ loop (Supplementary Fig. S8B). This is in stark contrast to the stem-loop RNA binding, wherein, a much larger interface in the RRM was involved. The only consensus, in both the cases, is provided by the residues Y274 and T275, which show strong CSPs (greater than averaged CSP + 2σ) on binding with either of RNAs. The lack of continuous clusters along with very few residues showing significant CSPs in the RRM implies a subdued interaction with the linear GGUG motif. A couple of biophysical studies have been carried out on the RRM of FUS and yielded contrasting insights on recognition of the linear GGUG motif. While both studies were extensively based on solution NMR analysis, in the first report, the RRM domain of FUS was titrated against an RNA containing the GGUG sequence and no observable interaction was found^20^. In a second recent report, the same RRM domain of FUS could recognize a GGUG containing 12-mer RNA with a *K*_*d*_ of 132-260 μM^30^. However, this reported *K*_*d*_ is 20–40 fold higher than the dissociation constants from our ITC analysis with *SON* stem-loop RNA indicating a 20–40 fold higher affinity for stem-loop RNA, further strengthening the importance of secondary structural elements in the RNA in mediating recognition.

The RRM domains of TAF15 and FUS are highly similar, with a sequence similarity of >95% and a RMS deviation of ~0.6 Å between both the structures. Therefore, it is logical to assume similar RNA targets and similar binding preferences for both the RRMs of TAF15 and FUS, based on the aforementioned similarities and broadly considering the non-specific nature of RRM-RNA interactions. However, most of reported residues and regions in FUS-RRM (which are as well as identical in TAF15-RRM) binding to GGUG containing 12-mer RNA showed no binding in TAF15-RRM against GGUG containing 6-mer RNA. Further, the unique expanded *α*_*1*_*-β*_*2*_ loop (Loop L2) of TAF15-RRM remained unperturbed, which was showing significant CSPs in FUS-RRM upon binding to GGUG containing RNA^30^. Detailed analysis into the binding residues of FUS-RRM has provided a surprising correlation. Most of the residues showing significant CSPs in FUS-RRM upon binding with 12-mer GGUG containing RNA are highly similar to the residues in TAF15-RRM showing significant CSPs upon binding with stem-loop RNA. These observed discrepancies and contradictions aided by the aforementioned correlation can be explained by the possibilities of GGUG containing 12-mer RNA used in binding studies of FUS-RRM forming stable secondary or higher order structures in solution, leading to observed CSPs which is otherwise not seen in the case of TAF15-RRM. Also, the lower or non-specific binding activity of TAF15-RRM toward GGUG RNA motif is bolstered by a similar previous study involving FUS^20^.

HITS-CLIP experiments have identified *in-vivo* RNA binding sites of TAF15 and a battery of best scoring RNA targets of different lengths reveals a recurring CUG motif^27^. Considering the spatio-temporal role-played by TAF15 in RNA processing events, such diverse functions seem to be a norm. In an attempt to understand the binding preferences toward CUG containing RNAs, varying lengths of RNA sequences were independently probed for binding using solution-state NMR spectroscopy. Surprisingly, the best 6-mer hit 5′ CCUCUG 3′, from the HITS-CLIP profile showed weak binding with the RRM domain of TAF15. Only seven residues in the RRM showed significant CSPs (greater than averaged CSP plus 1σ) upon binding to the RNA manifesting as a part of the C-termini (A318-R320) and the *β*_*2*_*-β*_*3*_ loop (Y274-T275) (Supplementary Fig. S8C, D). Similarly, another 7-mer RNA containing single CUG, 5′ GGCUGCG 3′, showed similar binding profile for TAF15-RRM (Supplementary Fig. S8E, F). To ascertain the possibility of a second tandem CUG repeat influencing RNA binding by the RRM, a 7-mer RNA containing two tandem repeats of CUG, 5′ GCUGCUG 3′, was analyzed for binding. The CSPs observed on binding to a 7-mer RNA containing two tandem CUG repeats were similar to those observed for 6-mer and 7-mer RNA binding containing single CUG (Supplementary Fig. S8G,H). These results clearly indicate that the RRM domain of TAF15 does not recognize CUG containing ssRNAs. These observed discrepancies from the HITS-CLIP profile can either be explained by insufficient primary sequence analysis of RNA which does not take into account of RNA secondary structural elements or the possibility of the RGG motifs and the RanBP2 type zinc finger domains playing a larger role in recognizing linear CUG containing RNAs, as evidenced by one of such similar studies^21^. An indiscrete analysis of all the CSP histograms of TAF15-RRM upon binding to various RNAs divulges an interesting commonality pertaining to the C-terminal region and the proximal part of *β*_*2*_*-β*_*3*_ loop comprising the residues Y274, T275 and to a lesser extent L273, T319 and R320, which shows significant CSPs. This indicates that these residues play a critical role in RNA recognition, but do not confer specificity.

## Conclusion

RNA-binding specificities of the FET family of protein have ostensibly, been widely spread out over several sequence and secondary structure motifs as evidenced by past studies. Our study provides a biophysical basis to this observation, centered on the interaction of RRM domain of TAF15 with *SON* stem-loop RNA and several CUG and GGUG containing linear ssRNA oligomers. Titrations of RNA against the RRM in solution have enabled us to narrow down to the *SON* stem-loop RNA as the most likely binding partner of TAF15 and FUS, given that other RNAs show little or no association *in vitro*. Also, we show that the *SON* stem-loop RNA recognition is predominantly mediated by the RRM with little or no influence by the downstream RGG and ZnF domains. Structural analysis of NMR structure of TAF15-RRM alone and its experimentally derived docked model with *SON* RNA has also provided sufficient evidence of a largely hydrogen-bonding mediated interface, which is in contrast to canonical RRM-RNA interactions that mainly involve amino acid-nitrogen base π-π stacking. While our docked model has provided crucial input regarding the non-canonical nature of RNA binding by this RRM, the mechanistic insights into base recognition and discrimination still remains elusive. This study, therefore, provides a platform for understanding mechanism of RNA recognition particularly by TAF15, and in general the FET family of proteins, which is likely to aid in understanding the molecular basis of neuromuscular disease progression.

## Methods

### Cloning, expression and purification of TAF15 and FUS constructs

The c-DNA clones of full length human TAF15 and FUS were obtained from DNASU plasmid repository (http://dnasu.org/DNASU/Home.do)^40^. TAF15-RRM (S231-E323) and TAF15-RRM-RanBP2 (S231-R388) constructs were cloned, over-expressed and purified as previously described^32^. Identical cloning, over-expression and purification methodology was used for preparation of FUS-RRM (S282-D374) and FUS-RRM-RanBP2 (S282-G454) constructs. All labeled proteins were prepared in the same manner.

### Selection of RNA sequences and preparation of *SON* stem-loop RNA

High scoring RNA sequence targets for TAF15 and FUS were selected based on the published literature involving biochemical, SELEX and CLIP experiments^27^,^29^. RNA oligonucleotides used in these studies (Table 3) were chemically synthesized and were obtained in 2′-deprotected and desalted form (Dharmacon Inc., USA). The RNA was dissolved in DEPC treated water and 5 mM or 10 mM stocks were prepared. *SON* stem-loop RNA was prepared by heating the stock solution of chemically synthesized RNA at 368 K for 2 min followed by immediate cooling on ice for 30 min to enable the formation of stem-loop.

### Nuclear Magnetic Resonance spectroscopy

The 3D ^13^C-edited [^1^H,^1^H]-NOESY and 3D ^15^N-edited [^1^H,^1^H]-NOESY spectra (NOESY mixing time = 100 ms) measured on 1 mM *U*-^13^C,^15^N-labeled TAF15-RRM in 20 mM sodium phosphate buffer pH 6.2, 50 mM NaCl, 5% D_2_O (*v/v*), 0.02% NaN_3_ (*w/v*) were used for structure calculation. Unlabeled *SON* stem-loop RNA was used at a concentration of 1.5 mM for homonuclear and heteronuclear experiments. For NMR titration experiments, the corresponding unlabeled RNAs were titrated against the 0.5 mM *U*-^15^N-labeled proteins at increasing molar equivalents of the RNAs until no further changes in the 2D [^15^N,^1^H] HSQC spectrum were observed, signifying saturation. The final NMR samples contained 1:1.2 stoichiometric complexes of 0.5 or 1 mM *U*-^15^N or *U*-^13^C,^15^N-labeled TAF15-RRM/FUS-RRM and corresponding unlabeled RNA in 20 mM sodium phosphate buffer pH 6.2, 25 mM NaCl, 5% D_2_O (*v/v*), and 0.5 or 1 mM *U*-^15^N or *U*-^13^C,^15^N or *U*-^2^H,^13^C,^15^N-labeled TAF15-RRM-RANBP2 and corresponding unlabeled RNA in 20 mM sodium phosphate buffer pH 6.2, 25 mM Na_2_SO_4_ and 5 mM *β*-mercaptoethanol. Buffers were prepared in DEPC-treated water. The NH backbone resonances of TAF15 in complex were unambiguously assigned using 3D HNCA, 3D HNCACB, 3D CBCAcoNH and 3D HNCO spectra^41^. Partial sequence-specific resonance assignments of *SON* stem-loop RNA were carried out at 273 K and subsequently at 298 K using 2D [^1^H,^1^H] NOESY (*t*_mix_ = 60, 150, 250, 350 ms) in H_2_O and D_2_O, 2D [^1^H,^1^H] TOCSY (*t*_mix_ = 50 ms) in D_2_O, 2D [^15^N,^1^H] HSQC in H_2_O and 2D [^13^C,^1^H] HSQC spectra in D_2_O at natural abundance. Two separate 2D [^13^C,^1^H] HSQC spectra were measured with frequency offsets and ^1^*J*_HC_ coupling constants optimized for base and sugar H^C^ resonances, respectively. All NMR spectra were measured on Bruker *Avance III* spectrometers equipped with 5 mm cryogenic triple resonance TCI probes, operating at field strengths of 500 and 700 MHz. All spectra were referenced to DSS^42^, processed with Topspin 2.1 (Bruker AG) and data was analyzed using CARA^43^. To identify the possible binding interface on the protein interface upon RNA binding, chemical shift perturbations (CSP) were calculated from the unbound and bound 2D [^15^N,^1^H] HSQC spectra using the following equation^44^;


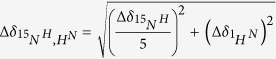


where Δ*δ*(^1^H^N^) and Δ*δ*(^15^N) are the changes in backbone amide chemical shifts for ^1^H^N^ and ^15^N respectively upon RNA binding. For determining regions of the RNA involved in interaction with TAF15-RRM, 2D ^13^C-[ω_1_,ω_2_]-filtered [^1^H,^1^H] NOESY (*t*_mix_ = 350 ms) spectrum in D_2_O and 2D [^1^H,^1^H] NOESY (*t*_mix_ = 60, 350 ms) spectra in H_2_O and D_2_O were acquired at 298 K for *SON* stem-loop RNA in complex with *U*-^13^C,^15^N TAF15-RRM in a 1:1 stoichiometric ratio. 2D [^1^H,^1^H] NOESY (*t*_mix_ = 60, 350 ms) spectra of the ^15^N labeled TAF15-RRM protein in D_2_O were also measured as a reference.

### Isothermal titration calorimetry (ITC)

All ITC experiments were carried out at 298 K on a Microcal iTC_200_ (GE healthcare) calorimeter. TAF15-RRM and FUS-RRM were prepared in 10 mM HEPES pH 7.0, 20 mM Na_2_SO_4_ while TAF15-RRM-RanBP2 and FUS-RRM-RanBP2 were prepared in 10 mM HEPES pH 7.0, 20 mM Na_2_SO_4_ and 5 mM *β*-mercaptoethanol. Use of DEPC treated double distilled water and buffers eliminated RNase contamination. 10-fold higher concentrations (1 mM in DEPC treated water) of various RNAs were titrated against 100 μM aforementioned protein constructs in the cell chamber with a stirring speed of 1000 rpm and 1 μl or 2 μl of regularly spaced (150 sec) sequential injections. The heat of dilution control (i.e. titration of RNA into buffer) was subtracted from the integrated peak area and the binding constant (K_a_), molar binding stoichiometry (N), molar binding entropy change (ΔS), and molar binding enthalpy change (ΔH) were determined directly from the fitted curve. Curve fitting and data analysis were carried out using the software Origin 7 supplied with the calorimeter.

### Structure determination of TAF15-RRM and analysis of backbone dynamics

We previously reported the sequence-specific resonance assignments of TAF15-RRM and TAF15-RRM-RanBP2^32^. Automated NOEs peak picking in all four 3D NOESY spectra and their assignments were performed using ATNOS^45^ and CANDID^46^ algorithms from UNIO software suite. Solution structure was calculated using CYANA 2.1^47^. UNIO’10 protocol was used wherein seven cycles of NOE assignments and structure calculations were iteratively performed which resulted in gradual reduction of target function scores^48^. Using 1537 NOE distance restraints and 487 dihedral restraints, a final ensemble of 20 structures was sorted from 200 calculated structures on the basis of RMSD. Dihedral angle (*φ* and *ψ*) restraints were obtained from the backbone and ^13^C^β^ chemical-shift values using the program TALOS-N^49^. H-bond restraints were obtained from H/D exchange experiments. The final solution structure ensemble was subjected to energy minimization in explicit water using algorithms from CNS^50^. The model was validated using PROCHECK^51^. Structural analysis and figure preparation were carried out using the molecular graphics packages, UCSF-Chimera^52^ and PyMOL. For the protein backbone dynamics, steady state ^15^N-{^1^H} nuclear Overhauser enhancements (NOEs) ratios were calculated using signal intensities from NOE and reference spectra (I_NOE_/I_ref_).

### Modeling of protein-RNA complex

Preliminary coordinates of *SON* stem-loop RNA were generated *ab initio* using Assemble2^53^ plugin within Chimera, employing 8 base pairs in the stem and 9 unpaired bases in the loop. The model was energy minimized using GROMACS^54^ within a hydrated dodecahedron of dimensions (7.1 × 7.1 × 7.1 nm^3^) containing 8008 explicit water molecules and Na^+^ and Cl^-^ ions up to a concentration of 150 mM. The AMBER99SB-ILDN^55^ force field was employed. The final model used for docking was obtained after running a production MD for 10 ns at 300 K in an NPT ensemble and selecting the best representative model from the largest cluster (Supplementary Data 1).

Experimentally-restrained docking of the modeled RNA on to TAF15-RRM was carried out on the High Ambiguity Driven Protein-Protein Docking (HADDOCK) web server^56^ (http://haddock.science.uu.nl/services/HADDOCK/haddock.php) using CSP information derived from NMR titration experiments. ‘Active residues’ were defined as those exhibiting perturbations greater than one standard deviation from the mean and ‘passive residues’ as amino acids exposed more than 50% to solvent in the vicinity of active residues. Given that existing structures of RRM-stem-loop RNA complexes involve binding to the RNA loop region (PDB IDs 1FJE, 2FY1 and 4PKD), bases of the *SON* loop (A11-A14) were defined as active residues with the passive residues being left as default. The lowest energy cluster representative was chosen as the docked model.

Since RNA coordinates used for docking in HADDOCK assume a largely rigid backbone, we performed simulation MD runs on the representative complex structure in GROMACS. Using the AMBER99SB-ILDN force field, the complex was solvated, neutralized and minimized in a cubic box (8.7 × 8.7 × 8.7 nm^3^) containing 150 mM NaCl and 20964 explicit water molecules. Following 100 ps rigid-body equilibrations in constant volume and constant pressure ensembles, a production run of 10 ns was performed at 300 K. The trajectories were clustered and the representative structure of the largest cluster (Supplementary Data 2) was used as the final model of the complex for analysis. Protein-RNA interface analyses were carried out using UCSF Chimera^52^ and PISA^57^ (http://www.ebi.ac.uk/pdbe/pisa/).

## Additional Information

**Accession codes:** Coordinate of the Human TATA binding protein associated factor 2 N (TAF15) has been deposited in the Protein Data Bank (PDB) under the accession code 2MMY. The sequence-specific resonance assignments of TAF15-RRM and TAF15-RRM-RanBP2 have been deposited in the BioMagResBank database (www.bmrb.wisc.edu) with the accession numbers, 19320 and 19561 respectively.

**How to cite this article**: Kashyap, M. *et al*. Structural delineation of stem-loop RNA binding by human TAF15 protein. *Sci. Rep*. **5**, 17298; doi: 10.1038/srep17298 (2015).

## Supplementary Material

Supplementary Information

## Figures and Tables

**Table 1:** NMR structure calculation statistics for the TAF15-RRM domain.

**Number of residues**	**97 (231-323)**
Number of models	20
Target function [Å^2^]	10.12 ± 0.67
Setup-given RMSD range [residues]-Backbone RMSD [Å]-Heavy atom RMSD [Å]	1–970.8 ± 0.311.2 ± 0.25
Optimal RMSD range [residues]-Backbone RMSD [Å]-Heavy atom RMSD [Å]	231–276, 280-3230.3 ± 0.140.7 ± 0.17
NOE restraints (upper) [#]-intraresidual (|i–j| = 0)-sequential (|i–j| = 1)-medium-range (1<|i–j|<5)-long-range (|i–j|>4)	1537277 (18.02%)481 (31.29%)253 (16.46%)526 (34.22%)
NOE restraints per residue	15.85
RMS NOE restraint violation [Å]	0.0384
Dihedral restraints [#]	487
RMS dihedral restraint violation [^ο^]	1.15
CNS energies [kcal/mol]-Total-van der Waals-Electrostatic	−2776.6−532.1>−3733.8
Ramachandran statistics*-most favored [%]-additionally favored [%]-generously favored [%]-disallowed [%]	87.911.80.2 0.1

*Derived from PROCHECK.

**Table 2:** Thermodynamic parameters derived from ITC measurements of TAF15 and FUS constructs with *SON* stem-loop RNA at 298 K.

**Construct**	**N**	**ΔH (Kcal/mol)**	**(ΔS (cal mol^**−1**^ **K**^**−1**^)**	**Kd (μM)**
TAF15-RRM	0.72 ± 0.01	−6.64 ± 0.15	0.5	10 ± 1
TAF15-RRM RanBP2	0.8 ± 0.01	−13.0 ± 0.30	−19.5	6 ± 0.7
FUS-RRM	N1*	−11.72 ± 1.06	−16.4	10 ± 1
	N2*	18.96 ± 1.89	86.0	13 ± 1.6
	N3*	−10.18 ± 2.12	−11.5	12 ± 0.3
FUS-RRM-RanBP2	0.7 ± 0.01	−13.03 ± 0.37	−20.3	8 ± 0.7

*Indicates sequential binding sites.

**Table 3:** RNA oligonucleotides used in these studies.

**RNA**	**Length**	**Sequence**
*SON* stem-loop RNA	25-mer	5′ CGUAUCUUUAACUACUCAAGAUACG 3′
GGU motif	6-mer	5′ GGUGUG 3′
CUG motif	6-mer	5′ CCUCUG 3′
CUG motif	7-mer	5′ GGCUGCG 3′
CUG motif	7-mer	5′ GCUGCUG 3′
