# Supplementary material for: Structural delineation of stem-loop RNA binding by human TAF15 protein
Source: Sci Rep. 2015 Nov 27;5:17298. doi: 10.1038/srep17298 (PMC4661536; doi:10.1038/srep17298)
Supplement: Supplementary Information [file srep17298-s1.pdf]

Structural delineation of stem-loop RNA binding by human TAF15 protein

Maruthi Kashyap[#], *Akshay Kumar Ganguly and Neel Sarovar Bhavesh*[*]

International Centre for Genetic Engineering and Biotechnology (ICGEB),

Aruna Asaf Ali Marg, New Delhi, India – 110 067

[*]To whom correspondence should be addressed. E-mail: neelsb@icgeb.res.in

[#]Present address: Inserm U869, Institut Européen de Chimie et Biologie (IECB), University of Bordeaux, 2 rue Robert Escarpit, 33607, Pessac, France

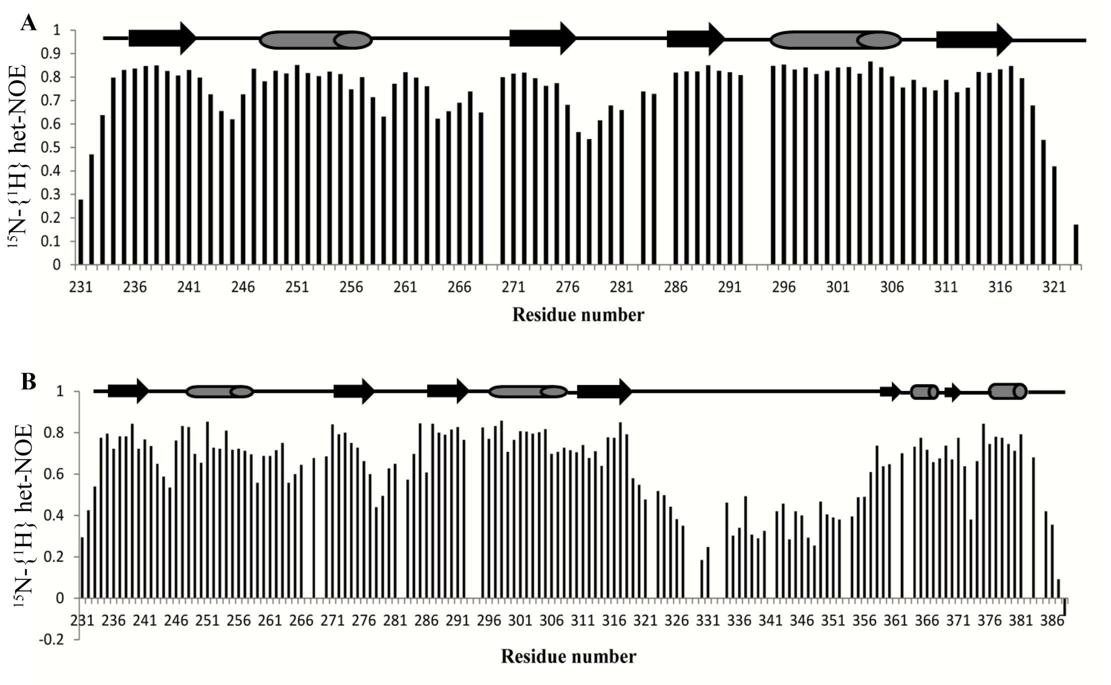

Supplementary figure S1. ^{15}N-{^{1}H} heteronuclear NOEs of TAF15 showing **(A)** highly structured RRM punctuated by few dynamic regions and **(B)** dynamic RGG motif linker between the structured RRM and RanBP2 type ZnF domains. The secondary structural elements are indicated on top (thick arrows indicate *β*-strands, cylinders indicate the α-helices and the loops and the disordered regions are indicated by thick line segments).

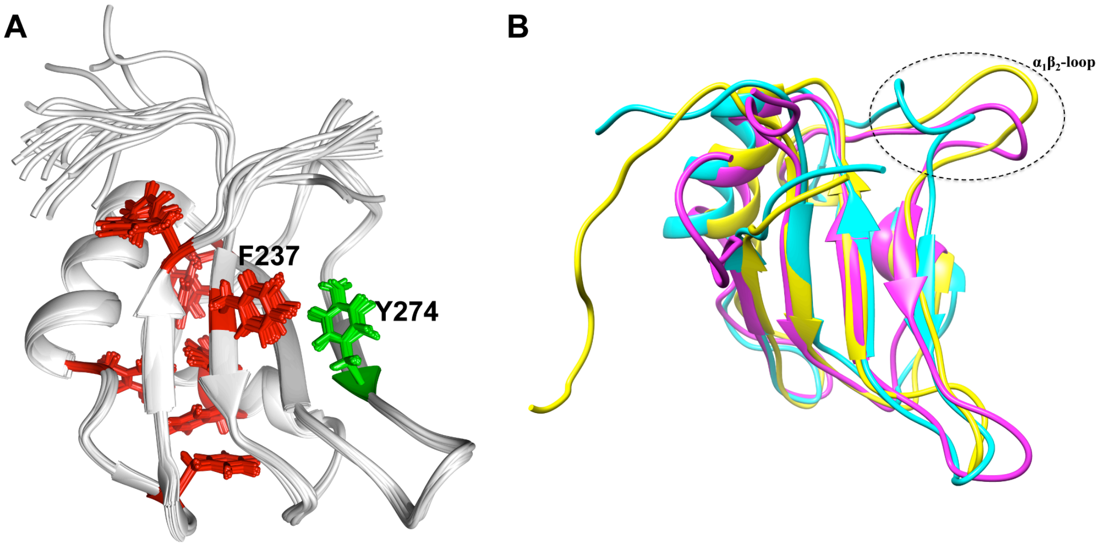

Supplementary figure S2. (A) Distribution of aromatic residues on TAF15 showing phenylalanines (red) and a single tyrosine (green). F237 and Y274 are the only surface-exposed aromatic residues on the *β*-sheet. **(B)** Structural alignment of TAF15-RRM (cyan) with FUS-RRM (magenta), PDB ID 2LCW and EWS-RRM (yellow), PDB ID 2CPE.

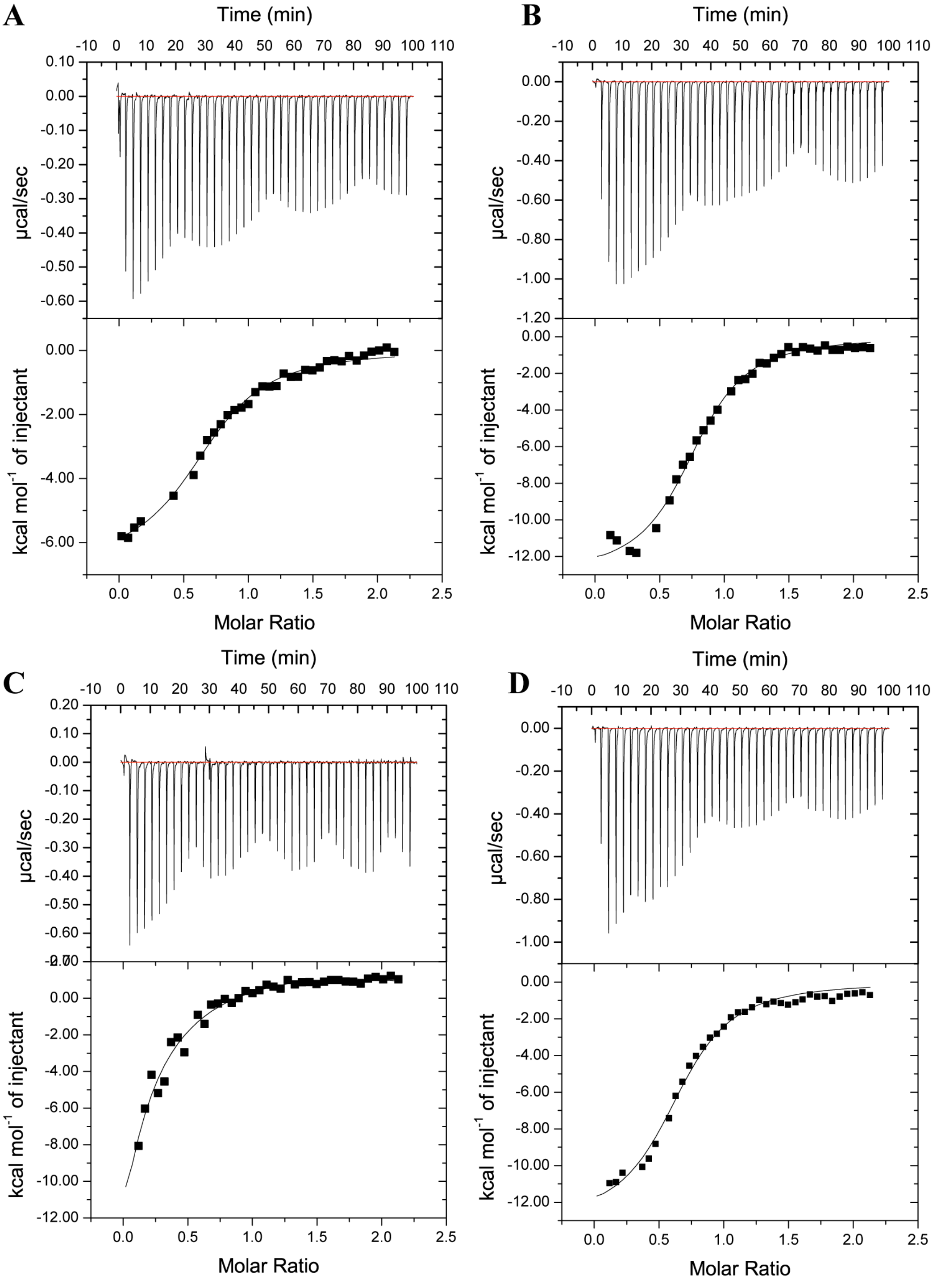

Supplementary figure S3. Binding isotherms and non-linear curve fitting for the titration of 1 mM *SON* stem-loop RNA in 100 µM solutions of **(A)** TAF15-RRM **(B)** TAF15-RRM-RanBP2 **(C)** FUS-RRM and **(D)** FUS-RRM-RanBP2 protein constructs.

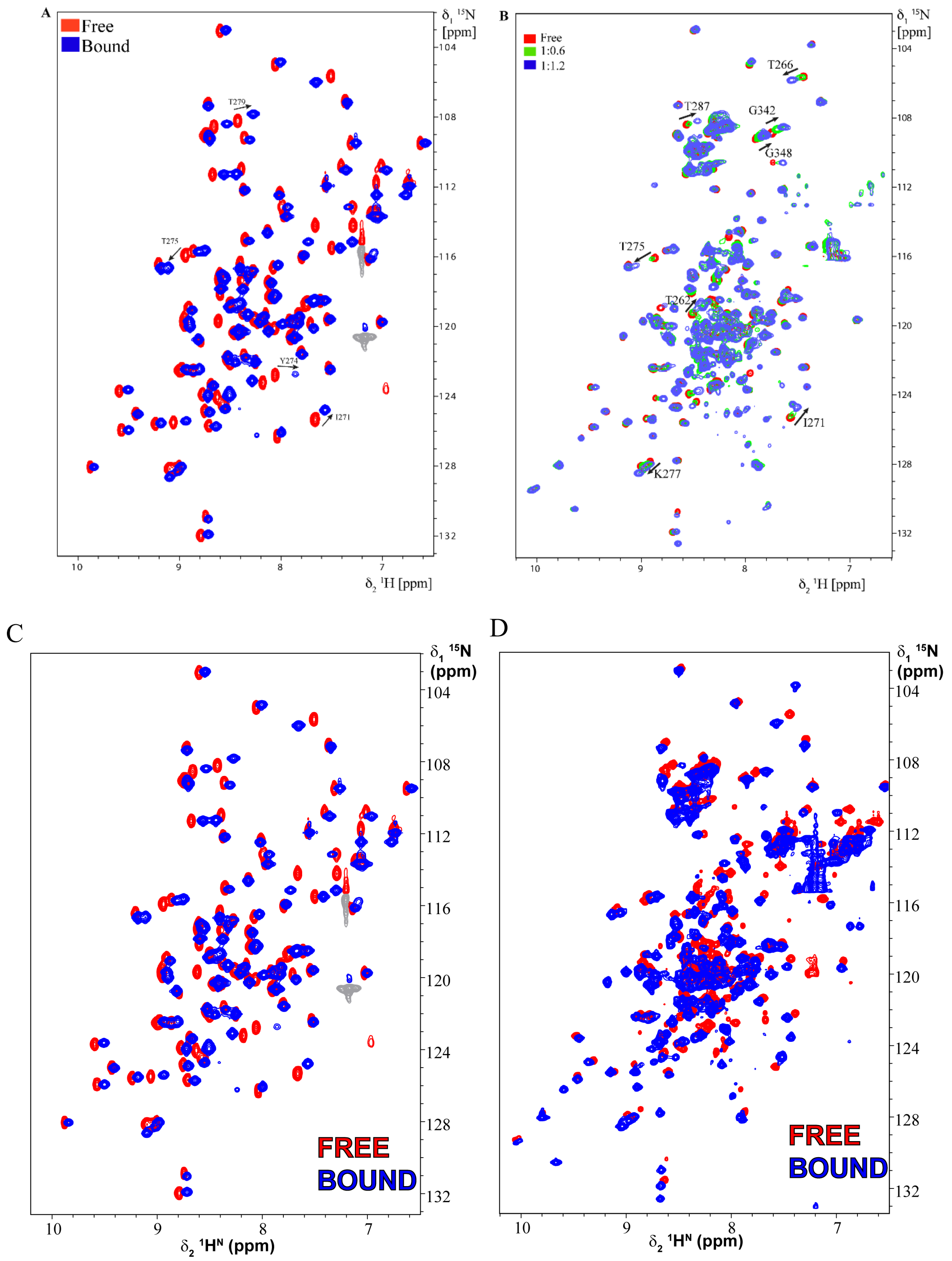

Supplementary figure S4: (A) Overlay of 2D [^{15}N,^{1}H] HSQC spectra of unbound and 1:1.2 bound TAF15-RRM with *SON* stem-loop RNA measured at 298 K. **(B)** 2D [^{15}N,^{1}H] TROSY spectra of TAF15-RRM-RanBP2 showing the progression of peaks upon titration with increasing molar stoichiometry (1:0.6 and 1:1.2) of *SON* stem-loop RNA measured at 298 K. CSPs for the same are shown in Fig. 2A (RRM) and 2C (RRM-RanBP2). (**C**) Overlay of 2D [^{15}N,^{1}H] HSQC spectra of unbound and 1:1.2 bound TAF15-RRM with *SON* stem-loop RNA measured at 298 K. (**D**) Overlay of 2D [^{15}N,^{1}H] HSQC spectra of unbound and 1:1.2 bound TAF15-RRM-RanBP2 with *SON* stem-loop RNA measured at 298 K.

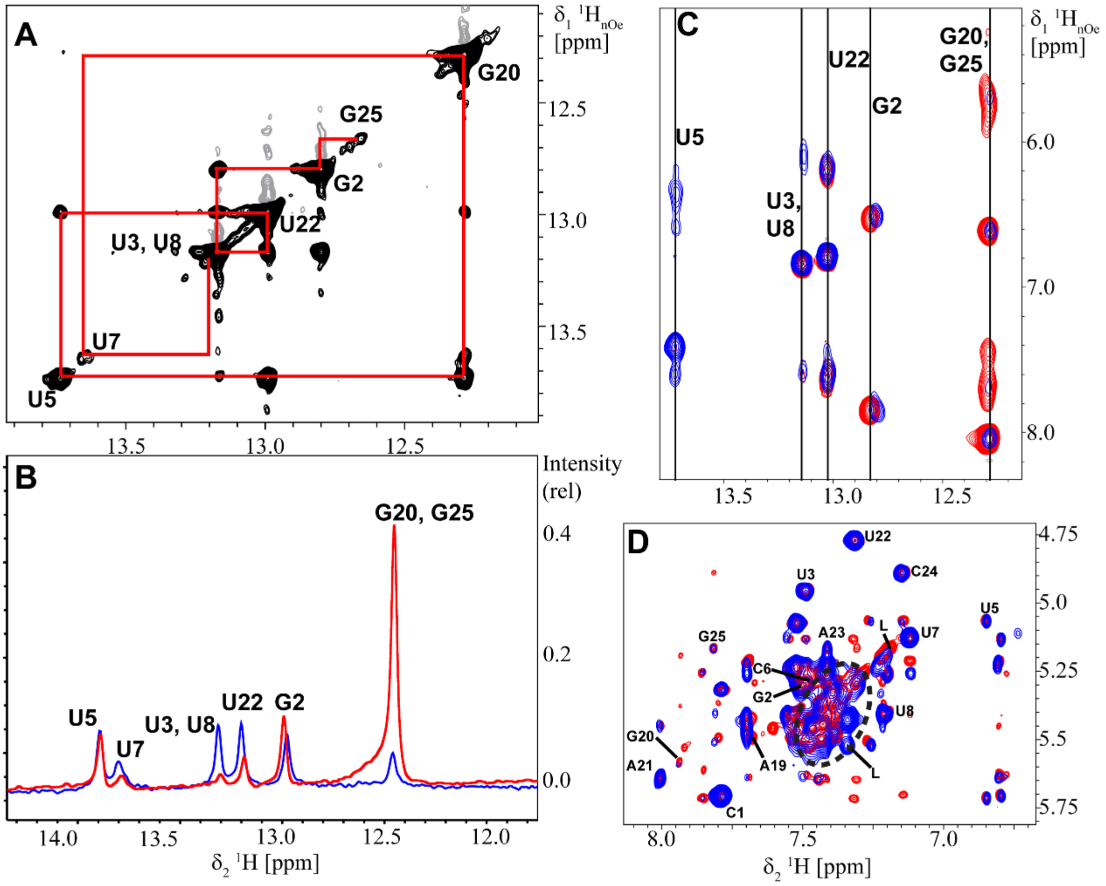

Supplementary figure S5. (A) 2D [^{1}H,^{1}H]-NOESY spectrum (t_{mix} = 350 ms) of *SON* stem-loop RNA in H_2O at 273 K depicting a sequential 'walk' across uracil H3 and guanine H1 resonances of the RNA stem. **(B)** Overlay of 1D ^{1}H spectra for free (red) and TAF15-RRM-bound *SON* stem-loop RNA (blue) in H_2O at 298 K with imino resonances labeled. **(C)** Overlay of 2D [^{1}H,^{1}H] NOESY spectra (t_{mix} = 350 ms) in H_2O at 298 K of free (red) *SON* RNA and complex of *U*-^{13}C,^{15}N TAF15-RRM with unlabeled RNA (blue) showing imino cross-peaks with base H^{Aro}/H^{Amino} and sugar protons. **(D)** Overlay of 2D [^{1}H,^{1}H] NOESY spectrum of free (red) *SON* RNA and 2D ^{13}C-[ω_1, ω_2]-filtered [^{1}H,^{1}H] NOESY (t_{mix}=350 ms) in D_2O at 298 K of *U*-^{13}C,^{15}N TAF15-RRM with unlabeled RNA (blue) showing changes in H^8-$H^{1'}$ (A/G) and H^6-H^5 (C/U) cross-peaks. Peaks arising from RNA loop are denoted by the letter 'L'.

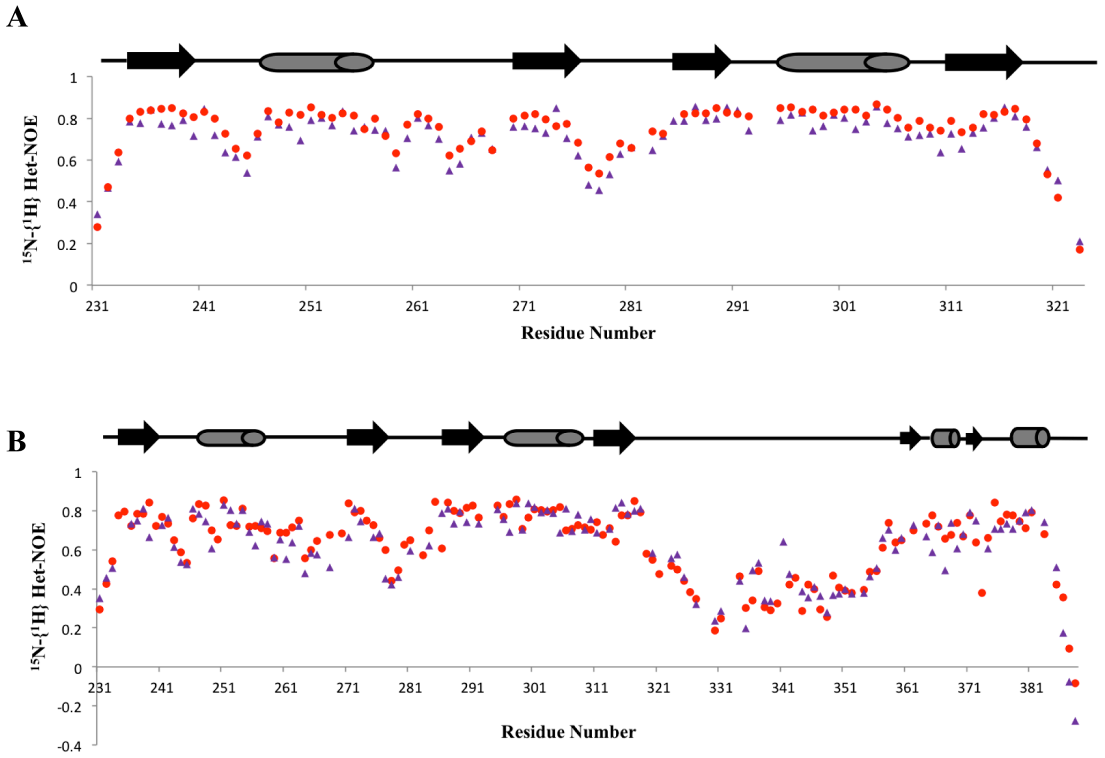

Supplementary figure S6. Comparison of ^{15}N-{^{1}H} heteronuclear NOEs of **(A)** TAF15-RRM and **(B)** TAF15-RRM-RanBP2 upon binding to *SON* stem-loop RNA. Red filled circles indicate the free protein constructs while blue filled triangles indicate their bound counterparts.

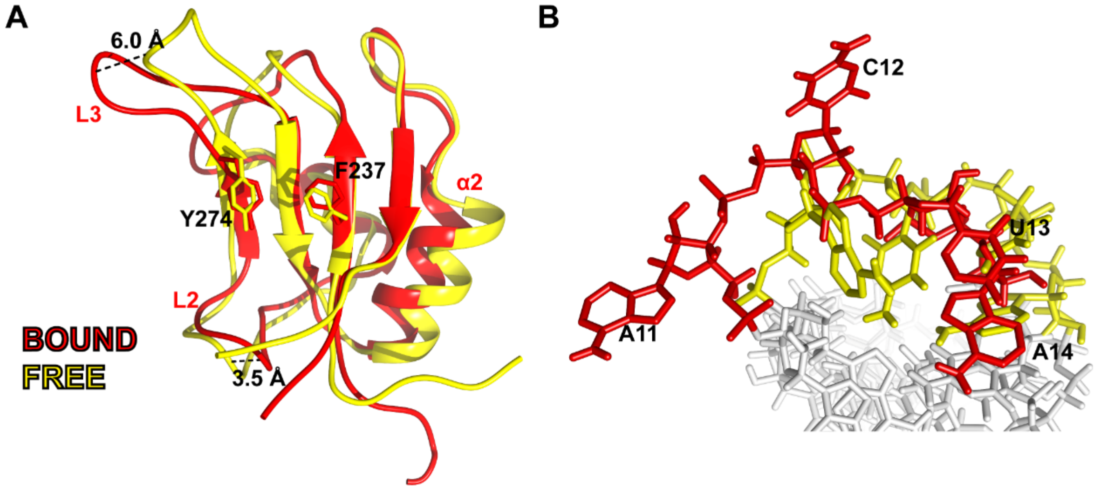

Supplementary figure S7: Structural rearrangements in TAF15-RRM and *SON* stem-loop RNA on binding. While the RRM shows movements of α_2 helix, loops L2 and L3 with a rearrangement of Y274 and F237 **(A)**, the RNA shows the adoption of a more extended state **(B)**.

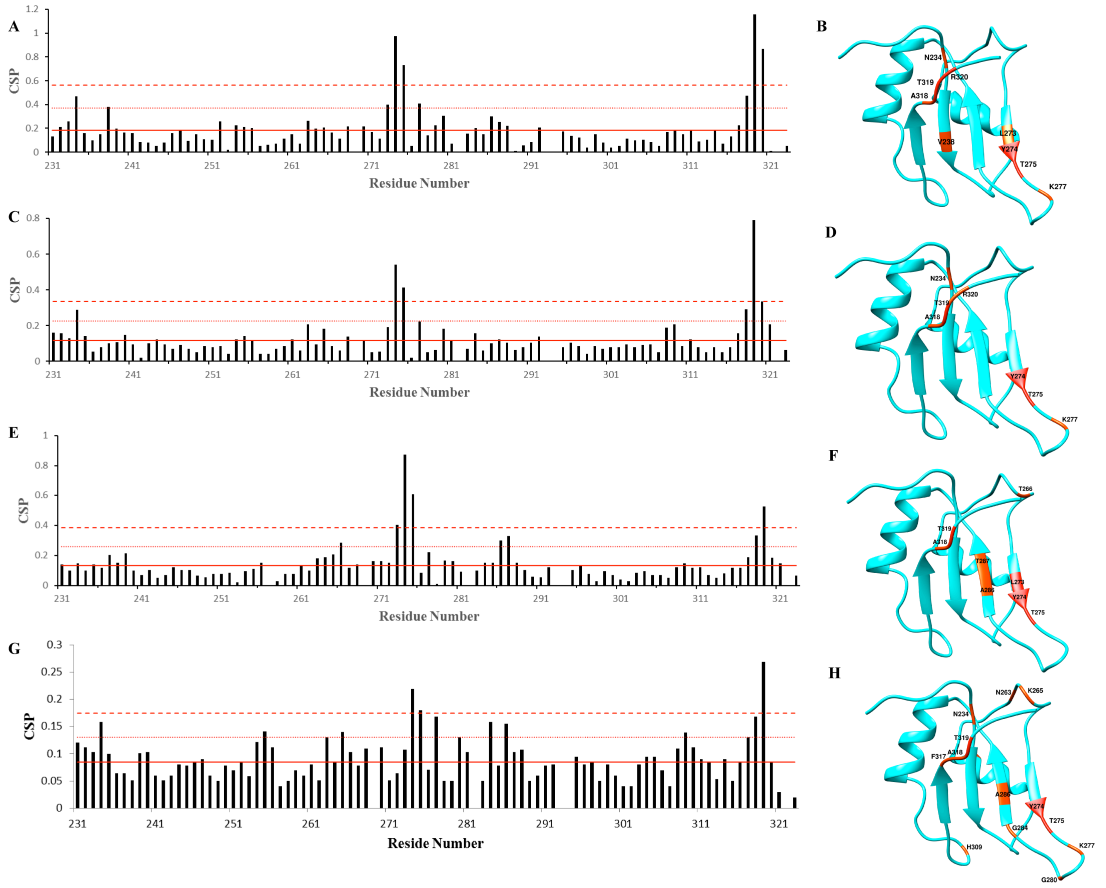

Supplementary figure S8: Chemical shift perturbation histograms of TAF15-RRM upon binding to 1.2 molar equivalents of **(A)** 5’ GGUGUG 3’, **(C)** 5’ CCUCUG 3’, **(E)** 5’ GGCUGCG 3’ and **(G)** 5’ GCUGCUG 3’ RNAs. The solid, dotted and dashed horizontal red lines indicate averaged CSP, one and two standard deviations from the averaged CSP respectively. The panels, **B**, **D**, **F** and **H** show CSPs mapped on to the solution structure of TAF15-RRM of corresponding histograms **A**, **C**, **E** and **G**. Residues exhibiting CSPs are colored in a gradation of red to orange-red. The color scheme is; orange red and red for one and two standard deviations from the averaged CSP respectively.
